# Magnetic-Assisted Treatment of Liver Fibrosis

**DOI:** 10.3390/cells8101279

**Published:** 2019-10-19

**Authors:** Kateryna Levada, Alexander Omelyanchik, Valeria Rodionova, Ralf Weiskirchen, Matthias Bartneck

**Affiliations:** 1 Institute of Physics, Mathematics and Information Technology, Immanuel Kant Baltic Federal University, 236016 Kaliningrad, Russia; kateryna.levada@gmail.com (K.L.); asomelyanchik@kantiana.ru (A.O.); valeriarodionova@gmail.com (V.R.); 2 National University of Science and Technology “MISiS”, 119049 Moscow, Russia; 3 Institute of Molecular Pathobiochemistry, Experimental Gene Therapy and Clinical Chemistry, RWTH University Hospital Aachen, D-52074 Aachen, Germany; rweiskirchen@ukaachen.de; 4 Department of Medicine III, Medical Faculty, RWTH Aachen, D-52074 Aachen, Germany

**Keywords:** liver fibrosis, magnetic fields, nanomedicines, immune cells, macrophages, hepatic stellate cells, RNA-based medicines, drug delivery, magnetic nanoparticles

## Abstract

Chronic liver injury can be induced by viruses, toxins, cellular activation, and metabolic dysregulation and can lead to liver fibrosis. Hepatic fibrosis still remains a major burden on the global health systems. Nonalcoholic fatty liver disease (NAFLD) and nonalcoholic steatohepatitis (NASH) are considered the main cause of liver fibrosis. Hepatic stellate cells are key targets in antifibrotic treatment, but selective engagement of these cells is an unresolved issue. Current strategies for antifibrotic drugs, which are at the critical stage 3 clinical trials, target metabolic regulation, immune cell activation, and cell death. Here, we report on the critical factors for liver fibrosis, and on prospective novel drugs, which might soon enter the market. Apart from the current clinical trials, novel perspectives for anti-fibrotic treatment may arise from magnetic particles and controlled magnetic forces in various different fields. Magnetic-assisted techniques can, for instance, enable cell engineering and cell therapy to fight cancer, might enable to control the shape or orientation of single cells or tissues mechanically. Furthermore, magnetic forces may improve localized drug delivery mediated by magnetism-induced conformational changes, and they may also enhance non-invasive imaging applications.

## 1. Introduction

The liver has a unique capability for regeneration, which has been known since Greek mythology. Strikingly, up to 70% of healthy liver tissue loss can be regenerated by its cells [1]. Regardless of the part, the liver of Prometheus regenerated overnight [1]. In evolutionary terms, the liver is the only organ in mammals that has preserved a high potential for regeneration to be replaceable after injury [2]. Despite this unique role, liver diseases are becoming an increasing burden of the health system. There are currently three stage 3 clinical trials with promising data. Future developments may include cell-selective targeting of key cell types of fibrogenesis, such as hepatic stellate cells (HSC). Here, we discuss magnetic-assisted applications including microfluidics technology, which have broadly enriched cancer therapy, including for instance in leukocyte engineering, i.e., in generating chimeric antigen receptor T (CAR T) cells. Microfluidic technologies have enabled the use of magnetic fields to control cell isolation, motility and directed migration, and modulating mechanical forces may also improve the methods to manipulate single cells. Medical applications of amplifying the precision of drug delivery towards tumor or dying cells at inflammatory sites are urgently needed. Directed use of magnetism may also further improve non-invasive imaging methodologies.

### 1.1. Liver Fibrosis

The capacity of the liver for regeneration is unique, but repeated and chronic liver injury frequently results in liver fibrosis. Fibrosis, which often precedes cancer, is characterized by the continuous accumulation of extracellular matrix (ECM), which is extremely rich in collagen I and III, leads to the deposition of scars and progressing on liver fibrosis [3]. This disease is characterized by an excessive accumulation of extracellular matrix (ECM) in the space of Disse. The accumulation of ECM has a negative effect on diverse functions of the organ such as detoxification and other liver functions, and it disturbs the hepatic blood flow. The recruitment of inflammatory immune cells, which can also amplify tumor development, represents another key event of fibrosis [4,5]. Untreated liver fibrosis can develop into cirrhosis and is accompanied by portal hypertension, hepatic encephalopathy, liver failure, and also is associated with an increased risk for the development of hepatocellular carcinoma (HCC) [6,7]. Liver injury usually is initiated by a noxa, virtually anything which can harm or kill the sensitive hepatocytes. Disease factors are viral hepatitis, chronic alcohol abuse, cholestatic disorders, genetic heritage, and autoimmune diseases. Apparently, nonalcoholic fatty liver disease (NAFLD) and nonalcoholic steatohepatitis (NASH) represent the major etiology of liver fibrosis. The demographic change caused by the ageing population and the growing epidemic of obesity lead to increased prevalence of liver fibrosis [8]. NAFLD is regarded as the main inducer of chronic liver disease in industrialized countries. It is assumed that NAFLD will be the leading indication for liver transplantation [9]. A significant number of as much as 20–30% of adults have NAFLD. Additional factors in disease, particularly immune cell infiltration, can lead to the progression of NAFLD to NASH and fibrosis. Fibrosis severity has been linked to mortality related to hepatic and other diseases, as evidenced in several longitudinal clinical studies and correspondingly, the effectiveness for the evaluation of drugs against NAFLD is their impact on liver fibrosis [9], which may also have a positive outcome on nonhepatic diseases [10]. It was estimated that liver-related mortality will increase dramatically in the next decade [9]. Fibrosis can be considered a dysregulated wound-healing response which leads to scarring of tissues. Different disease etiologies exhibit specific hallmarks, but advanced stages are commonly characterized by bridging fibers between portal fields [11].

### 1.2. Roles of Different Hepatic Cell Types in Liver Fibrosis

The process of fibrosis development, fibrogenesis, can be analyzed from a cellular perspective. The regeneration of hepatocytes works in a streaming fashion, as shown in a rat model by Zajicek and colleagues in 1985. Hepatocytes located at the portal space gradually stream towards the hepatic vein where they are eliminated by apoptosis. This cellular traveling was estimated to last 201 days in rats [2]. However, during liver fibrosis, the empty spaces left by the missing hepatocytes are frequently replenished with ECM by HSC, rather than with fresh hepatocytes. During the course of cell death, certain molecules are released by hepatocytes, which function as danger signals for other cell types, for instance for HSC [12]. These “alarm bells” also attract immune cells which themselves secrete pathogenic factors that can induce apoptosis of hepatocytes. These processes result in an amplification of the fibrogenic response. Innate immune cells are well known to initiate liver inflammation in NAFLD and they express pattern recognition receptors (PRR) which can sense danger-associated and pathogen-associated molecular pattern molecules (DAMP and PAMP), as well as inflammatory mediators [12].

In recent years, the roles of cholangiocytes, particularly in cholestatic liver injury, started to be explored. For instance, cholangiocyte proliferation is a substantial driver of liver fibrosis in biliary atresia. Researchers demonstrated that a long non-coding RNA has a major impact on the proliferation of cholangiocytes and thus represents a therapeutic target in this regard [13]. Damage of cholangiocytes in toxic liver injury leads to a hampered production of bile acids. Sato and colleagues further revealed that extracellular vesicles and microRNAs might be critical factors which regulate cyclic adenosine monophosphate (cAMP) metabolism in cholangiocytes [14].

Mast cells are another cell type reported to further amplify hepatic fibrosis and injury. Particularly, mast cell-deficient mice exhibited less pronounced fibrosis. It was reported that mast cells regulate the proliferation of cholangiocytes and contribute to the activation of HSC [15]. A means to specific deactivation of mast cells might therefore represent a novel therapeutic strategy. However, the involvement of mast cells in fibrosis remains controversial since they have been reported to be both harmful and protective [16].

The hepatic macrophages form a complex mixture of cells of various activation stages and cellular origin. They can be distinguished as resident macrophages, which were originally defined as Kupffer cells, as well as monocyte-derived macrophages (MoMF). These cells can be separated using cell sorting applications [17]. Kupffer cells were first discovered by Karl Wilhelm von Kupffer in 1876 [18], even before the relevance of phagocytic cells was first published by Metchnikoff in 1888 in Tuberculosis [19]. While cell sorting has unraveled hepatic macrophage subpopulations that were characterized in RNA bulk sequencing where thousands of cells are analyzed in a single RNA isolate [17], single cell RNA sequencing has begun to start unraveling the real complexity of hepatic macrophage subtypes [20]. Single cell RNA sequencing has also enabled identification of subpopulations of HSC [21]. The so-called resting or quiescent HSC (qHSC) form a homogenous population characterized by high platelet-derived growth factor receptor β (PDGFRβ) expression. However, the activated HSC, which are also called myofibroblasts, can further be sub-divided into populations expressing α-smooth muscle actin (α-SMA), collagens, or immunological markers. The S100 calcium binding protein A6 (S100A6) was identified as a universal marker of activated HSC, myofibroblasts (MFB), for both mRNA and protein expression [21]. The so-called transdifferentiation of the resting and vitamin A storing HSC into MFB, which are proliferative and which express huge amounts of collagen, is central for fibrogenesis [22]. The activation of HSC can be induced through a variety of extracellular signals from other liver cell types like hepatocytes and macrophages. Further, intracellular processes like oxidative stress, autophagy, endoplasmatic reticulum stress, or metabolic dysregulations, have been studied in great detail and are regarded as causative for HSC activation [23]. The fact that fibrosis is a bidirectional path is evidenced by reports on fibrosis regression [24]. HSC thus represent a valuable target for fibrosis therapy since they are the source of excessive matrix production. Inhibiting the construction of different collagens, which in part takes place in the extracellular space by specific inhibitors, for example, Lysyl oxidase-like 2 (LOXL2), has recently been proposed to be done by small molecules [25].

**Figure 1:**
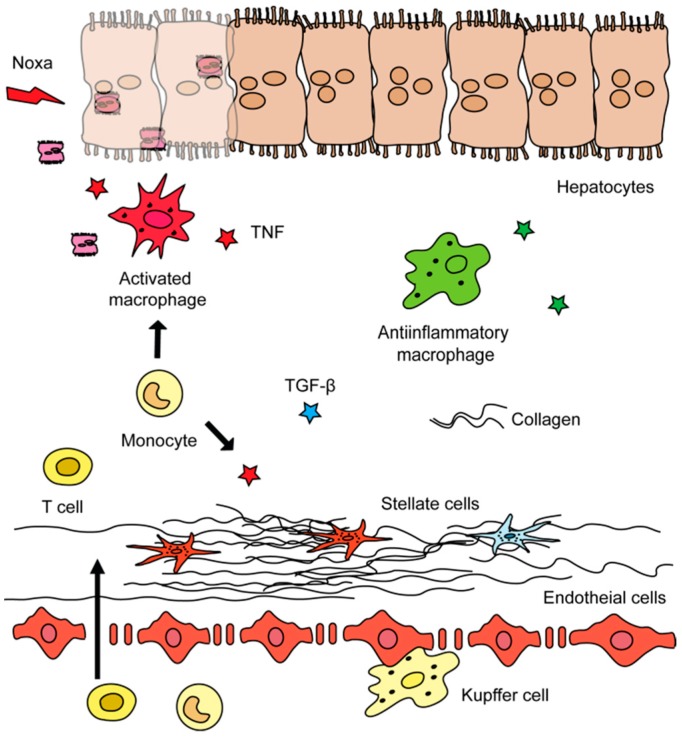
Cells, their roles, and potential targets in liver fibrosis. Liver disease is in most cases initiated by a noxa that leads to hepatocyte cell death. Cytokines secreted by immune and other cell types promote hepatocyte cell injury, i.e., the tumor necrosis factor (TNF) triggers apoptosis of hepatocytes. Hepatic collagen deposition by activated hepatic stellate cells is a hallmark of fibrosis and in part is facilitated by extracellular enzymes.

It was shown that, if a circumvention of the inflammatory insult is achieved, among other factors, the anti-inflammatory and restorative activities of macrophages persist and impact the hepatic microenvironment [26]. During fibrosis regression, MFB are eliminated through cell death induction, become senescent, or may even revert into cells which resemble quiescent HSC [27]. The excessively deposited ECM was shown to be degraded, i.e., by matrix metalloproteinases (MMP) that are released by certain subtypes of macrophages [24] (Figure 1).

### 1.3. Current Clinical Trials on Liver Fibrosis

Nowadays, the treatment options for liver fibrosis remain scarce and the most efficient strategy remains to overcome the vicious circle of liver injury. This results in an eradication of viruses or weight reduction and dietary changes in NAFLD, in order to stop disease progression or in the ideal case, by inducing fibrosis regression [28,29,30]. Currently, three stage 3 clinical trials are ongoing, which will be ready in the next years. Importantly, none of these focus on the direct modification of the pathogenic mechanisms of fibrosis, but on other mechanistic links such as the cause for the underlying liver injury, signals from other organs, for instance, derived from the intestine (i.e., bile acids), or from immune cells, metabolic activation, or cell death. The cell death of hepatocytes represents a key mechanism for liver fibrosis [31]. The inhibition of cell death has emerged as a therapeutic strategy in the treatment of liver fibrosis [32]. The classical definition of cell death separates necrosis (uncontrolled death of cells, autolysis) from apoptosis (regulated cell death). However, it has become apparent that many types of necrosis in fact are specifically regulated, and the terms necroptosis, ferroptosis, or autophagy have been brought up [33]. However, a general inhibition of cell death might be problematic because apoptosis of HSC is also required in fibrosis regression [23,31]. Furthermore, cell death induction is also required to trigger the death of cancer cells. During oncogenesis, cancer cells can overcome apoptosis to escape from elimination by immune cells. Many candidate drugs have been developed to interfere with the development or progression of hepatic fibrogenesis (Table 1).

An ideal treatment strategy would be cell-type specific, and, in the case of targeting cell death, should be hepatocyte-specific. In line with these conclusions, the ASK1 inhibitor Selonsertib (GS-4997, Gilead), which has been studied in NASH patients in a stage 3 clinical trial (STELLAR 4) has not been successful. Selonsertib exerts its activity by inhibiting an important cell death switch of Apoptosis-signal-regulating kinase 1 (ASK1). ASK1 is induced by oxidative stress and enhances hepatocyte death, inflammation, and fibrosis [34]. However, there is still new hope from three other stage 3 clinical trials on liver fibrosis. Targeting another facet of the disease, two drugs target hepatic metabolism: the drug obeticholic acid (OCA) and Elafibranor (ELA). OCA is a synthetic lipophilic bile acid which is already approved for the treatment of primary biliary cholangitis (PBC). OCA acts via activating the nuclear bile acid receptor Farnesoid X receptor (FXR), and this in turn leads to a reduction of bile acids (which are produced in the liver and the small intestine. OCA is a semi-synthetic analogue of natural bile acids and exhibits a more than 100-fold increased stimulation of FXR compared to the natural bile acids. OCA leads to reduced bile acid production, and also to a reduction in the uptake of glucose and lipids from food [35]. This drug is currently tested in a huge phase III trial in NASH patients (REGENERATE). The drug ELA is an Insulin-sensitizer which aims to improve the action of natural insulin. ELA activates Peroxisome-proliferator-activated receptors (PPAR). The concept of ELA is to prevent fat deposition in hepatocytes and thereby steatosis, by removing glucose from circulation. It acts on (PPARα/δ R) via agonism. The phase III trial on NASH patients is performed by the company Genfit (RESOLVE-IT). There are also attempts to combine both OCA and ELA, which further improve liver histology in fibrosis models [36].

While OCA and ELA act on metabolism, Cenicriviroc (CVC), another small molecule, blocks the two important chemokine receptors CC chemokine receptor 2 (CCR2) and CCR5. The result is that it blocks recruitment of lymphocytes and monocytes. It exerts anti-fibrotic activity in animal models [37]. The CENTAUR study was the first clinical study, which employed an orally available drug in a prospective study [38]. Currently, the AURORA study, which includes a phase III trial, is running using NASH patients (Tobira Therapeutics, NCT03028740).

There is a growing list of promising phase I and II trials that include potential novel directions. Similar to Selonsertib, other drugs aim to prevent cell death and it is envisioned that this can be reached via inhibition of caspases, major regulators of cell death. Examples for this are given by Emricasan (Conatus, phase II), VX-166 from Vertex, and Nivocasan (GS-9450) by Gilead (phase I/II).

The metabolism is being targeted most intensively. Similar to OCA, Tropifexor (Novartis, phase II), Cilofexor (GS-9674, Gilead, phase II), AKN-083 (Allergan, phase I/II), and partially, also INT-767 (Intercept, phase I/II) target the FXR metabolism. Aramchol (Galmed, phase II) is a different bile acid/fatty acid conjugate. Similar to ELA, Saroglitazar (Zydus Cadila, phase II trial) and Lanifibranor (Inventiva, stage II trial) target PPAR signaling, targeting different ligands than ELA. Saroglitazar is directed towards PPARα/γ while Lanifibranor to PPARα/γ/δ.

Artificial PEGylated fibroblast growth factor (FGF) 21, BMS-986036 imitates the functions of the liver-derived hormone FGF21 that regulates the activation of fatty acids [39], which is now in stage II trials. Insulin signaling is modulated by agonists of the glucagon-like peptide-1 (GLP-1), i.e., Liraglutide or Semaglutide (both Novo Nordisk, phase II). These drugs exert their beneficial effects by improving insulin resistance, inducing weight loss, and ameliorating NASH [40]. The synthesis of lipids in the liver is blocked by inhibitors of Acetyl-coenzyme A carboxylase such as GS-0976 (Gilead) or PF-05221304 (Pfizer) (both stage II clinical trials) [41]. Inhibitors of acetyl-coenzyme A carboxylase (ACC inhibitors), like GS-0976 (Gilead) or PF-05221304 (Pfizer), also reduce hepatic lipogenesis and reduce hepatic fibrosis [41]. Mimicking signals from the gut represent an additional option to target the liver. The gut-derived hormone FGF19 in humans (FGF15 in mice), among others, regulates bile acid synthesis. Therefore, an FGF19 analogue has been generated to treat fibrosis (NGM282, NGMBio), which reduces the hepatic fat content [42].

## 2. Theoretical Background of Magnetism

In order to basically understand the potential which controlling magnetic forces might offer for novel treatment options for liver fibrosis, it is important to comprehensively understand nanoscale magnetism. Nano-sized particles are often used as tools in this regard since they can specifically target cells or structures in the body. Magnetic nanoparticles (MNPs) usually refer to ferro- or ferrimagnetic crystals sized below 100 nm [43,44,45]. At the nanoscale, many physical properties, including the short- and long-range magnetic interactions, contribute to the overall functionality of these nanoparticles [46]. The magnetic material is characterized by a strong response under an action of the magnetic field. The investigation of a family of 3D nanocrystals has been growing with impressive speed over the past decades. Among them, the MNPs are an exciting class of material for biomedical applications [43,47,48,49,50]. Magnetism in matter is related to spins − the smallest magnetic units referred to as atoms are composed of ferro(i-)magnets, which interact via a quantum phenomenon called the exchange interaction that leads to the formation of long-range ordered areas (magnetic domains) [44,46]. Owing to the superposition character of the magnetic field, the total magnetic moment of this area is equal to the sum of individual moments (µ) of each atom. If a magnetic field with the strength H is applied, the magnetic moments prefer to align along the direction of the magnetic field to reduce the total energy (by the domain walls movement in the macroscopic body). Thus, when the magnetic field is strong enough to align all individual magnetic moments, a ferromagnetic body is saturated, and the magnetic moment of this system equals N·µ, where N is the total number on the individual magnetic moments of the atoms in the system. For characterization of material mass or volume-weighted parameters in magnetization saturation, M_S_ = N·µ/m or N·µ/V (Am^2^/kg and A/m in SI, or emu/g and emu/cm^3^ in CGS). The behavior of magnetization has a hysteretic character for ferro(i-)magnetic materials because of specialties of the magnetization processes such as domain wall pinning on defects. Those irreversible processes lead to nonzero magnetization (M_R_) at the remnant state when the magnetic field is off. Intrinsic energy, which keeps the spins in a certain direction in the absence of a magnetic field, is called the magnetic anisotropy energy and it determines the hysteresis loop width or coercivity field µ_0_H_C_ (T in SI and Oe in CGS).

Macroscopic magnets tend to reduce their magnetic moments (or, in other words, to reduce their magnetostatic energy) and split into randomly oriented domains [44,45]. Magnetic domains are separated by domain walls − intermediate states are required to rotate spins in differently magnetized domains to reduce exchange interaction with the interface. When the size of MNPs is comparable with the size of domain walls, the split into domains is no more energetically favorable, and MNPs transform to the single-domain state. A single particle presents a saturated magnet with magnetization equal to the saturation magnetization value. In the ideal case, all spins below Curie temperature are oriented in one direction, and because of exchange interaction, their behavior can be described by the superposition of all spins. The orientation of this macrospin in the absence of a magnetic field is defined by easy axis−positions where total energy is minimal. The energy which separates the macrospin at a certain position is magnetic anisotropic energy (E_a_). That means that to change orientation along the axis, the system needs to overcome the energy barrier equal to E_a_ = K × V. Thus, particles of bigger volume (V) have higher anisotropy energy and the second term, K, is an anisotropic constant defined by material and structural-morphological properties of MNPs. Several sources contribute to anisotropy with constant K. Among them the more pronounced are the shape anisotropy arising from magnetostatic interactions of poles and the magnetocrystalline anisotropy coming from spin-orbital coupling. The behavior in the magnetic field of assembly of randomly oriented single-domain MNPs with one easy axis was described by Stoner and Wohlfarth in 1948 [51]. Magnetic properties of single-domain MNPs are different compared to bulk analogs – they may have higher values of remnant magnetization and coercivity field because the coherent rotation magnetization reversal mechanism is more difficult than the domain wall moving and additional contributions to anisotropy coming from the surface.

The range of diameters when MNPs pass in the single-domain state is 20–800 nm, for magnetic iron oxides (magnetite Fe_3_O_4_ or magnetite γ-Fe_2_O_3_) it is 80–90 nm [44,52,53]. For MNPs of smaller size, the magnetic anisotropy energy becomes comparable to the thermal energy K × V ≈ k_B_ × T, where k_B_ is the Boltzmann constant. In this regime, called superparamagnetic (SPM), the temperature induces random switching of magnetization of single MNPs, and thus the time-averaged M_R_ and H_C_ of MNPs assembly are zero [54]. The probability of this fluctuation is described by the Néel relaxation time:

(1) $$\tau_{N}=\tau_{0}e^{\frac{\mathrm{KV}}{k_{B}T}}\;$$

where τ_0_ is an attempt time ~10^−9^ s [44]. The temperature at which MNPs act in the SPM regime at a fixed observation time is the so-called blocking temperature T_B_. For quasi-static measurements the typical measuring time is 100 s and the blocking temperature can be evaluated as T_B_ = K × V/25k_B_. The fluctuating magnetic moment of MNPs with T_B_ less than the ambient temperature is favorable for their colloidal stability since it reduces dipolar interactions among them. Moreover, SPM MNPs do not change their magnetic properties when the viscosity of the medium is changed, for example, if MNPs are internalized by cells [55].

### 2.1. Magnetic Nanomedicines

Nanomedicines, nanotechnologically generated drugs, cover a broad range of sizes of a few up to several hundred nanometers [56]. Depending on the nature of the material, they can be classified into two major groups, organic or inorganic. Owing to their small size, nanoparticles can easily be dispersed in aqueous solutions, which is crucial for intravenous administration [57,58]. The nanoscale exhibits a high surface to volume ratio, meaning that the comparatively high surface can be functionalized with ligands. These ligands can change the pharmacokinetics of the particles, i.e., polyethylene glycol (PEG) can increase the circulation time of nanoparticles [58,59,60]. Specific ligands for cellular receptors can enable specific binding to specific cell types. Moreover, this valuable surface can be conjugated via drugs for their delivery and on-demand release [59,61], or with fluorescence marker and other molecules for multimodal imaging or therapy [62,63]. The prefix “magnetic” means that MNPs are sensitive to the magnetic field. This field brings numerous options that are attractive for biomedical applications since this field can easily penetrate the body and interact with MNPs, for example, for their detection, visualization, manipulation, or heating.

The magnetite Fe_3_O_4_ and magnetite γ-Fe_2_O_3_ are the most applicable magnetic materials in the biomedical field. Bulk magnetite has a cubic inverted spinel structure and exhibits a ferrimagnetic behavior at room temperature. The nano-sized magnetite oxidizes rapidly into maghemite, which also has similar ferrimagnetic properties and spinel structures with some valences. The iron oxide-based MNPs of a diameter (d) below 30 nm act in the SPM regime at room temperature and are abbreviated as SPIONs (superparamagnetic iron oxide nanoparticles) [53]. Particles sized less than 10 nm are usually attributed to ultra-small SPIONs (USPIONs [53]) and are characterized by reduced magnetization and increased anisotropy because of the influence of non-collinear spins at the surface [64,65]. The SPIONs and USPIONs are in the frame of interest for biomedical applications, especially for magnetic resonance imaging (MRI) where they are applied as contrast agents for both T1 and T2 relaxation times [66,67,68]. For specific applications, for instance, magnetic hyperthermia, the adjustable anisotropy of MNPs, is needed [69,70]. For this reason, magnetic anisotropy can be tuned, for instance, by variation of the chemical composition of ferrites (Me^2+^Fe_2_O_4_) by doping the spinel structure of ferrite with the ions of transition metals (Me^2+^ = Co, Mn, Zn, and others [71].

#### 2.1.1. Methods for Synthesis of Magnetic Nanomedicines

The various types of synthesis strategies for MNP can be separated into two approaches: top-down and bottom-up. The “bottom-up approach” starts from metal ions in solution via chemical methods and is probably the most commonly used strategy. The top-down approach starts with bulk material, which is further processed, i.e., by laser ablation [72,73,74,75,76] or lithography [77,78,79] including that of nanospheres [80]. The lithography techniques have broad control on the shape of nano or microstructures; however, the scaling of this method to large-scale production was reported to be challenging [81]. The laser ablation method offers the building of quite complicated structures such as core-shell MNPs and has a lot of degrees of freedom for adjustment by variation of the environment, material of target, laser regime, as well as external stimulus, for example, magnetic or electrical field to change the shape and structural properties of MNPs [72,75]. This method beats some drawbacks of more common chemical methods; for example, it does not require high temperature, pressure, or organometallic precursors to obtain MNPs with excellent magnetic properties [76,82]. Thus, the laser ablation method has great potential to set higher standards in nanoparticle production.

However, many methods are combinations of both types of methods. For example, the known ball milling method of the MNP preparation is popular for permanent magnet fabrication [83]. It allows one to scale up the synthesis to an industrial scale, although the control of particle shape and size is difficult. Potential agglomerations of nanoparticles can occur which makes the particles unsuitable for biomedical applications [83]. Nevertheless, in combination with chemistry, the ball milling equipment can be used in the so-called mechanochemical process. Here, for instance, a nanocomposite of MNPs in the benzene-1,3,5-tricarboxylic acid matrix was obtained via a mechanochemical process [84]. The obtained material was porous and defined as a metal-organic framework. This nanocomposite was tested for a drug delivery application to release doxorubicin as a model drug. The authors noted that the high surface area of such porous materials favors an increased loading rate, while the magnetic properties of this material offer novel perspectives for diagnostic systems [84].

Typical synthesis steps of chemical methods consist of different steps, particularly burst nucleation, and the following nanocrystal grow, which is called Ostwald ripening [85,86]. Control of reaction kinetics by varying temperature, solvent or other conditions, and operation with the Ostwald process by pH control and electrostatic repulsion of nuclei allow us to systematically vary the size of the particles [86]. It is of utmost importance to obtain nanoparticles with precise and predefined size, shape, and phase composition [87,88]. It was suggested to evaluate the most commonly used synthesis strategies by the four-word strengths, weaknesses, opportunities, and threats (SWOT) analysis for applications in molecular recognition [88]. The research group evaluated the co-precipitation, thermal decomposition (HTD), microemulsion, and microfluidic synthesis method and studied dual-particles consisting of several materials. The first one, co-precipitation, is an easy to use technique to obtain large amounts of MNPs by alkalization of metal salt solutions. First demonstrated in 1981 by Massart, this method is beneficial and allows us to produce well-crystallized iron oxide or ferrite MNPs in the size range of 10−30 nm [89,90,91]. The drawbacks of this method are the poor control of shape and size distribution; moreover, for smaller particles, less than 10 nm, the quality of crystals decreases and the number of disordered spins leads to a change in the magnetic properties [64,92]. Advanced co-precipitation methods are performed at high temperature and pressure, by hydrothermal surface treatment, or hydrothermal routes [93,94,95], as well as in non-aqueous medium by solvothermal methods [96,97,98]. The polyol process is another interesting method which is a cost-effective and easily scalable method to produce MNPs of high quality and variety morphology, from simple pseudo-spherical to multi-core nanoflowers of core-shell MNPs [99,100,101]. In the polyol process, solvents also play the role of a reducing agent and a surfactant.

Invented in 2004 for the synthesis of MNPs, the HTD method allows us to obtain MNPs with a narrow size distribution and high crystallinity [102,103,104]. MNPs produced with this method have a high value of magnetization, favoring their use in many biological applications, including sensors and detection [92,105]. According to the SWOT analysis above described [88], a drawback of this method is that it is time consuming and expensive. Precise shape control can be achieved by varying the experimental conditions. For instance, a variation of ligands and surfactants offers advantages for both magnetic properties and the related behavior in biological environments [87,106,107]. The group of Jinwoo Cheon [108] demonstrated higher magnetization values of cubic MNPs (165 emu/g_Fe+Zn_) compared to spherical (145 emu/g_Fe+Zn_) particles. The difference can be attributed to the lower amount of disordered spins on the surface. It was also reported that cubic shaped MNPs on the sensor‘s surface exhibit a higher binding ability because of the higher contact area of planar interface compared to the spherical one [106]. Furthermore, cubic MNPs exhibited stronger signals, as evaluated by giant magnetoresistive sensing (GMR) and force-induced remnant magnetization spectroscopy (FIRMS).

There are various methods for co-precipitation with different modifications, which, together with HTD, are the most frequently used methods for MNP synthesis [60,88]. Co-precipitation represents the most important production method: it is easy, cheap, and enables a rapid synthesis. The generated particles have hydrophilic surfaces, which can be functionalized in situ. The second most important method, HTD, allows one to produce MNPs with well-defined shape and narrow size distribution. However, the low amount of reaction products, high-cost reagents, and hydrophobic surfaces, which can be functionalized in the multi-step process, make this method currently mostly interesting only for research activity.

#### 2.1.2. Clinical Use and Further Perspectives Iron Oxide

Currently, Feraheme^®^ (ferumoxytol) which is approved by the U.S. Food and Drug Administration (FDA) as well as in Europe and Canada is the most successful iron oxide-based drug. It is prescribed to patients with iron deficiency anemia and chronic kidney disease. It can be considered as a great advantage that iron is a naturally occurring element of the body which also leaves the body via natural pathways of iron metabolism [109]. Feraheme is based on non-stoichiometric magnetite MNPs with diameter (d) ~ 7 nm and hydrodynamic diameter (d_H_) = 28–33 nm coated with carboxy-dextran [66]. Feraheme is also an ‘off label’ magnetic resonance angiography (MRA) and magnetic resonance imaging (MRI) contrast agent [66,110]. In order to study MNP uptake by macrophages in vivo, high-resolution 3D-maps of pancreatic inflammation were generated using MRI and it showed that the MNP uptake by macrophages was higher in the inflamed pancreatic lesion in T1D-models [111]. Therefore, the special properties of the inflammatory setting on noninvasive imaging have to be considered. The capability of iron oxide to produce reactive oxygen species (ROS) was recently discovered to be usable in order to treat leukaemia [112]. In contrast to intravenously administrated drugs, the oral delivery (OD) is more popular with patients. Yet, the passage of the gastrointestinal (GI) tract, which has a very low pH level, [113] is a problem for iron oxides since they degrade at this pH. Thus, to overcome this limitation, a coating is required which is stable in the wide pH range of 2–8. Coatings such as gold or silica oxide are applicable for this purpose since these materials are stable in the GI tract.

Mesenchymal stem cells (MSC) have been demonstrated to be a promising tool for the treatment of many types of human diseases, including liver fibrosis and hepatocellular carcinoma (HCC) [114,115,116,117,118]. Labeling of MCS with MNPs can be used as a supplementary diagnostic approach. Recently, Faidah at al., proved that application of MNPs have not changed the viability and proliferative capabilities of MSCs in a rat cirrhosis model based on a carbon tetrachloride (CCl_4_) model for toxic liver injury [119]. Additionally, similar results by Lai and co-authors have shown that MSCs that overexpress human hepatocyte growth factor (HGF) promote liver recovery in a rat liver fibrosis model [120]. Further, labeling of MSC with NP led to an accumulation of the cells in MRI-based imaging [119,120], which seems to confirm the idea that magnetic NPs in liver fibrosis can be used as a diagnostic agent for MRI.

The off-label use of feraheme is continuing to grow. The assessment of the stage of liver fibrosis remains a key issue in patient diagnostics. Currently, the gold standard is still given by a liver biopsy while imaging techniques as elastography and relaxometry have not been elaborated in this regard, particularly, for proving moderate fibrosis [121]. A recent study showed that patients with different stages of liver fibrosis exhibited MRI-assessible differences. One should, however, be aware that T2 parameters can be quantified via a dual echo turbo-spin echo technique. Besides, these applications can also help to understand different stages of fibrosis patients [121]. It is noticeable that T2 or negative contrast agents possess superparamagnetic properties and are represented by nanoparticles with iron oxide core or other magnetic materials [122]. For this purpose, researchers are using different types of synthesis, shapes and covering of nanoparticles with magnetic cores. One should, nevertheless, consider the associated problems from another perspective: Li and coworkers proposed to conjugate Fe_3_O_4_ NPs with immunofluorescence markers (indocyanine green (ICG). Further, a targeting ligand for integrin avb3, arginine–glycine–aspartic acid (RGD) expressed by HSCs to detect early stages of liver fibrosis, was used [123]. A similar approach was used by Zhang and coauthors, who investigated diagnostic of liver fibrosis stages in a rat model induced by CCl_4_ injection. Although they were using molecular MRI with RGD peptide, modified ultrasmall superparamagnetic iron oxide nanoparticles (USPIO) were demonstrated to be a promising tool for noninvasive imaging of the progression of the liver fibrosis [124].

Furthermore, one should note that together with the advantageous properties of magnetic materials which makes them usable as MRI contrast agents, the iron overload in tissues can be really harmful. Thus, Wei and coauthors reported that a single dose of MNP application at a high concentration (5 mg Fe/kg) induced a septic shock response at 24 h and provoked high levels of serum markers (ALT, AST, cholesterol and other markers), which was noted within 14 days. Moreover, a high dose of MNPs activated significant expression changes of a distinct subset of genes in cirrhosis liver compared with a normal dose of MNP application (0.5 mg Fe/kg) [125]. Furthermore, Lunov et. al. analyzed the impact of MNPs on macrophages and demonstrated that overload of iron leads to apoptosis in these cells, which is mediated via activation of the c-Jun N-terminal kinase (JNK) pathway [126]. This investigation demonstrated that iron overload caused by MNP application may have dramatic effects, particularly on the liver, which contains a large numbers of macrophages.

#### 2.1.3. Magnetic Hybrid Nanomaterials

Iron oxides exhibit moderate cytotoxicity; however, for their use in biomedicine, such aspects as biotransformation, biodistribution and in-blood circulating time should be controlled [60,127]. Because of this reason, the iron oxide MNPs can be hybridized, i.e., with certain polymers (dextran, chitosan, PEG) [128], noble metals (Au, Ag) [129,130], non-magnetic oxides (MgO, ZnO) [131,132], or silica dioxide (SiO_2_) [133]. Apart from polysaccharides, PEG is more often used for organic coating of MNPs. By varying the molecular weight of the PEG from 6000 to 50,000, it is possible to prolong the circulation time (blood half-life) from 30 min to one day [134]. The coating leads to a change in the surface charge of MNPs and, therefore, also significantly affects the pharmacokinetic behavior [60]. Typically, the negatively charged MNPs exhibit a longer blood half-life [60]. The coating also makes it easier to modify the surface for modifications with biomolecules, genes, or drugs. Furthermore, hybrid materials possess additional functionalities, for instance, nano-dimensional gold exhibits the surface plasmon resonance phenomenon changing the optical properties of the material, which can be exploited for the detection in photothermal therapy [129,135,136]. Maria Efremova and her colleagues reported on nanohybrids of magnetite and gold in the form of Janus-like MNPs [129]. Two district surfaces established a platform for conjugation with two different molecules, for example, with fluorescent dyes or drugs. These nanohybrids exhibited enhanced contrast for MRI and allowed tracking delivery of the attached drug in a real-time fashion via intravital fluorescent microscopy.

Lipid-based nanomedicines represent the nanomedicines with the highest market value. Via a combination of SPIONs embedded into the lipid membrane of liposomes, the desired properties of both materials can be combined. One example of an application with magnetoliposomes is the application of a magnetic field to induce release of nucleic acids, such as DNA, which might be useful in drug release. Salvatore et al. demonstrated that SPIONs can trigger release of DNA from a multifunctional hybrid nanomaterial composed of liposome components, double-stranded DNA, and hydrophobic SPIONs [137].

There is a huge and virtually an endless number of options for hybrid nanomaterials and we only shed light on these. Their numbers are very likely to further increase in future nanomedicines.

### 2.2. Magnetic Materials in Drug Delivery

#### 2.2.1. Magnetic-Assisted Medical Applications

The size of nanomaterials is a critical factor for their behavior and distribution in the body. Small particles in the blood tend to aggregate or to be decorated with a protein corona and, because of their charge, create an electrical double layer. In this sense, the total size of particles characterized by the hydrodynamic diameter (d_H_) can be measured i.e., using dynamic light scattering (DLS) [138]. Both the hydrodynamic and physical size of MNPs determine their magnetic properties. In circulation, systemically injected MNPs circulate in the lumen of blood vessels, interacting with macrophages of the reticuloendothelial system (RES). Smaller USPIONs with d_H_ > 10 nm were characterized by longer blood half-life than SPIONs d_H_ > 50 nm [66,134]. Depending on the hydrodynamic size as well as the electrical charge and other properties, pharmacokinetics and biodistribution of MNPs are rendered [60,139]. MNPs with a d_H_ below than 15 nm are filtered by the kidney; MNPs with a d_H_ less than 100 nm accumulate in the liver in hepatocytes and Disse space; a d_H_ of 100–150 nm leads to the primary accumulation in liver that is based on the uptake by Kupffer cells and these larger hydrodynamic particles can also be trapped by splenic macrophages.

Since the liver and spleen are primary targets for MPN accumulation, they can also be targeted with these particles. The hydrodynamic size of 10–50 nm seems optimal for longer circulation time, but it should be stressed that not only size matters, but properties of the surface such as zeta-potential and hydrophilic/hydrophobic properties are critical factors for biodistribution [60,134]. Non-specific biodistribution of MNPs was used for MRI enhancement of liver disease [139,140]. Ferrucci and Starkre reported that 80% of intravenously injected non-specific SPIONs were internalized by Kupffer cells. Thereby, the MNPs create an MR-contrast that enables us to trace hepatic neoplasms [140].

We would like to highlight four non-exhaustive areas in which magnetic materials are important (Table 2). The first area includes the biggest success story for using magnetic fields for binding-mediated cell capturing. The chimeric antigen receptor (CAR) T cells have significantly advanced tumor therapies. This technology is based on the isolation of T cells from autologous donors and these cells are genetically engineered to target a specific antigen. The isolation of T cells is currently approved for treatment of B cell lymphoma, and it is expected that T cell engineering will enrich many other fields as well; clinical studies are ongoing in liver cancer. The group of Michael Sadelain pioneered T cell engineering and they have recently shown that macrophages play a key role in mediating the side effects of CAR T cell therapy [141]. Other groups also attempt to manipulate other immune cells, such as natural killer cells [142]. Microfluidics that facilitate binding-mediated cell capturing and release is a key technology for sorting cells in closed systems [143,144,145]. The microfluidic concept for manufacturing lab-on-a-chip devices use MNPs and these allow for a quick analysis and an automated composition of an individual treatment. Currently, the CAR T cell therapies are mainly focused on B cell malignancies, but other types of cancer are being explored in clinical trials.

Tissue engineering has been given further opportunities by magnetic fields, i.e., by facilitating the arrangement of different cellular layers [146,147,148]. Magnetic fields have been used to establish three-dimenstional cell culture arrays, to enable cell patterning for the evaluation of the effect of fibroblasts on their capability of infiltration [149,150,151]. Rieck et al. recently demonstrated that magnetic nanocarriers can be localized in specific areas of the body using magnetic fields. Their approach was done using complexes of lentivirus and MNPs in combination with magnetic fields. The group highlighted that using this method in the murine embryonic stem cell system offers the opportunity to site-specifically downregulate protein tyrosine phosphatase SHP2 by RNAi technology in selected areas with the pathogenic vessel formation [152]. Using this principle would allow us to selectively transmit a specific payload to immune cells based on phagocytic uptake to diseased sites in the liver. Muthana et al. demonstrated that even magnetic resonance imaging machines might be usable to control the spatial concentration of MNPs [153].

The second field of application that we would like to emphasize is mechanical cell control. Here, the aim is to get control over the mechanical properties of cells. In the near future, the technology may be used to measure the stiffness of biological molecules or cellular organelles in vivo. Magnetic tweezers are used as a simple tool to study mechanical forces of biological molecules and cells [154]. Low-frequency magnetic fields are applicable for directed cell destruction and induction of apoptosis [78,155,156]. The concept of mechanical cell control has already been used to function as a cellular remote control, to modulate the stem cells differentiation, or to modulate the behavior of single cells [157,158,159]. Magnetic micromanipulation based on the application of magnetic tweezers is a potent biophysical technique that is applicable for single-molecule unfolding, rheology measurements, and analyses of force-regulated processes in living cells [160,161].

The third type of application is drug delivery. MNPs are a powerful tool for therapy to target killing of injured or infected cells, which may be achieved by, for example, native toxicity of the MNPs, thermomagnetic effect in magnetic hyperthermia, or targeted drug delivery and release [135,162,163]. In particular, the field of drug delivery can potentially be highly enriched by magnetism-based applications. In addition to the above-mentioned application of MRI scanners to spatially concentrate magnetic particles, magnetic fluids, meaning magnetic particles in a dispersion, might be controlled magnetically. The release of drugs, for example, mediated by magnetism-enhanced thermosensitive polymers, might also be enriched by magnetic actions, for example, to trigger the release of different drugs from a single carrier from porous materials, remote-controlled drug release using azo-functionalized iron oxide nanoparticles, or in order to trigger heat and drug release from magnetically controlled nanocarriers [133,164,165]. In addition to particulate or crystal-based systems, surface immobilized iron might be controlled using noninvasive magnetic stimulation [166,167,168,169].

The fourth area in which magnetism has been used intensively is applications based on imaging. For instance, MRI-based imaging of liver fibrosis can be enabled by visualizing fibrosis based on non-invasive analysis of collagen or elastin. Fibrosis might even be visualized by labeling HSC, i.e., by monitoring a specific surface marker such as the folate-receptor, that has already been used for this purpose. Folate receptor-targeted particles have also led to improved specificity of tissue binding [66,124,170]. In order to enable an early therapy of fibrosis, it is critical that it is also diagnosed at early stages. However, there is currently no established early stage marker. In the future, improved early recognition techniques such as circulating collagen fragments, i.e., the N-terminal propeptide of collagen III (Pro-C3) will very likely enable to perform “liquid biopsies” for early detection of fibrosis. Screening of Pro-C3 in plasma of patients has already been done in hepatitis C patients and has been demonstrated to be able to predict fibrosis progression [171]. Other biomarkers might be circulating micro-RNA, and other types of RNA, such as long non-coding RNA (LncRNA), epigenetic analyses, and microbiome studies. Usually, biomarkers work best if they are combined to calculate a specific score such as the NAFLD fibrosis score, the fibrosis 4 (FIB-4) index, or the aspartate aminotransferase to platelet ratio index (APRI) score.

Non-invasive imaging techniques have high potential to confirm the findings from biomarker screening. Consequently, there is a high unmet need for the development of improved techniques for non-invasive diagnosis of liver fibrosis [116]. In ref. [116], the molecular MRI was used for distinguishing different fibrosis stages in a CCI_4_-based rat model. Enhanced accuracy of detection was achieved by using a targeted USPIO-based contrast agent for MRI. The MRI contrast depends on the time of relaxation of protons that alters in the presence of a magnetic field generated by MNPs and it depends on the degree of interaction of the MNPs with protons. Furthermore, direct imaging of the distribution of MNPs is possible by magnetic particle imaging (MPI) introduced in 2005 and based on measurements of the nonlinear magnetic signal of MNPs [172,173]. Potentially, MPI can enable an improved spatial and temporal resolution than the resolution of the MRI and because of the native biodistribution of iron oxide-based MNPs, the use of this technique for liver seems promising. Improved detection methodologies may also further improve the usage of magnetic particles as biosensors [174]. Imaging probes such as those monitoring Elastin will very likely also improve imaging of liver fibrosis at later stages of the disease, as Elastin appears in late-stage fibrosis [175] (Table 2).

The tripeptide arginine-glycine-aspartic acid (RGD) binds to integrin αvβ3 expressed on HSCs [124]. Conjugation with RGD significantly improved targeting of administrated USPIONs [124]. This allowed authors of ref. [124] to differentiate various liver fibrosis stages with MRI. The relaxivity of developed nanoparticles was higher than, for example, the earlier reported collagen-specific contrast agent based on Gd^3+^. Further improvement was done by multimodal imaging with nanohybrids [123]. In this study, it was suggested that the conjugation of USPIONs-SiO_2_ with indocyanine green (ICG) dye may further improve near-infrared fluorescence imaging and RGD for targeting. This combination of imaging modalities enables it to perform multimodal imaging (NIR and MRI). To establish theranostic platforms, an additional drug should be attached [176,177].

**Figure 2:**
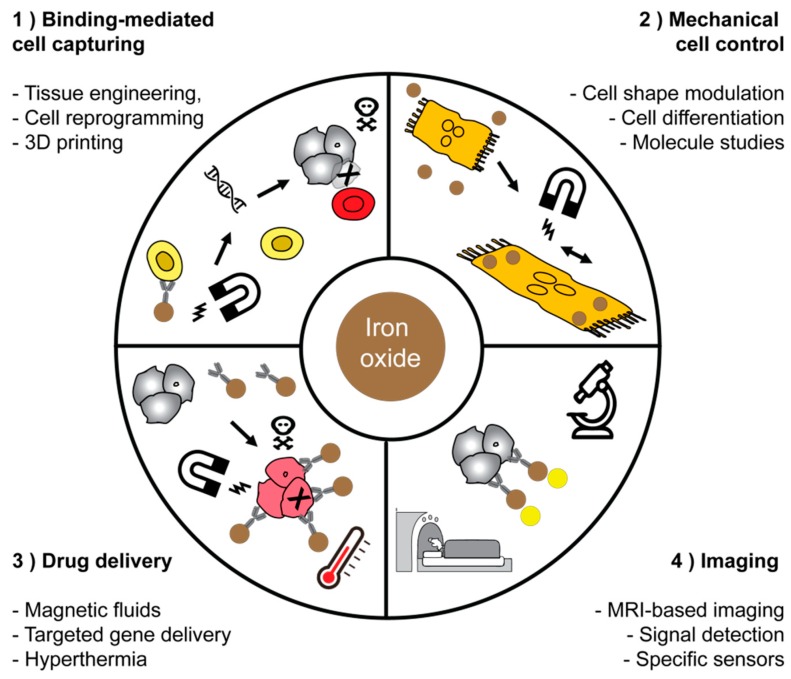
Applications of magnetic nanoparticles in medicine and biotechnology. We would like to highlight four main fields of application for magnetic materials and have chosen some representative schemes for each field of application.

In order to further visualize some selected applications of magnetic fields, we have illustrated these with a sketch. In particular, cell sorting technology which is currently enabling breakthroughs in cancer therapy is largely based on magnetic forces to sort cells, i.e., the magnetic-assisted cell sorting technology (MACS). These technologies are based on magnetic fields and enable sorting and manipulate cells in closed systems. Mechanical cell control is still in its infancy, but may significantly enrich single cell analysis methods. The field of drug delivery may assumingly expect high potential for breakthroughs due to the potential localized targeting of tissues or cells in vivo, to reduce side effects on non-target cells with enhanced specificity. Imaging applications are already profiting from steadily improved probes, improved methods for signal detection, and better biological targets. In fibrosis imaging, non-invasive assessment of Collagen and Elastin might in future enable significantly improved and more specific assessment of liver fibrosis (Figure 2).

#### 2.2.2. Selective Targeting of Hepatic Stellate Cells—Mission Impossible?

Cell type-specific treatment of disease was first proposed by Paul Ehrlich, who suggested the development of magic bullets which aim at the pathogens only [178]. Ehrlich assumed that people would in the near future be able to specifically target cells or tissues to cure disease with unmet specificity. However, such directed therapies for cancer have been challenged in the past with unexpected problems. One example of a company focusing on cell targeting is BIND therapeutics which went bankrupt in 2016 [179]. Through re-investments, its drug, encapsulated Docetaxel targeting the prostate-specific membrane antigen (PSA), has entered clinical testing again and is currently evaluated in a phase 2 trials in patients with metastatic castration-resistant prostate cancer [180]. It can be assumed that targeting liver fibrosis might face similar difficulties like cancer. However, without any doubt, HSCs as the key cells for fibrosis, represent an ideal target in this regard since they are the main source of activated myofibroblasts and portal fibroblasts that control the fibrogenic process [181].

However, reaching HSCs with drugs is intrinsically difficult since they are located in the perisinusoidal space between hepatocytes and sinusoidal endothelial cells. Under normal physiological conditions, one major function of qHSCs is a depository for vitamin A [182]. In response to liver damage, inflammatory mediators promote HSC activation and their subsequent differentiation into myofibroblasts [183]. Activated HSCs (aHSC) are the main source of collagen in the liver and can abundantly secrete ECM proteins, tissue inhibitors of metalloproteinases and matrix metalloproteinases (MMPs), which cause remodeling of the liver architecture [184]. It is important to note that HSCs are responsible for 80% of the total fibrillar collagen I in the fibrous liver [185]. Thus, inactivation or modulation of HSC is a one of the key aspects in the development of innovative fibrosis therapy.

HSC and MFB express/upregulate diverse specific receptors, such as the mannose-6-phosphate/insulin-like growth factor II receptor (M6P/IGFII) [186,187], PPAR [188], integrins [189], platelet-derived growth factor receptor (PDGFR) [181], or a peptide receptor of the relaxin 1 family (RXFP1) [182]. Various receptors of HSC have been engaged with specific drugs directed to these. The mannose 6-phosphate/Insulin-like growth factor receptor (M6P/IGFR) is among the most popular targeting structures for HSC. The strategy of eliminating the HSC was investigated in a study 2006 by Greupink et al., in which a nanocarrier consisting of human serum albumin (HSA) was used as a carrier, and functionalized with mannose-6-phosphate (M6P) for this purpose. In order to eliminate these cells, the authors employed doxorubicin, which is used in cancer therapy to kill tumor cells [190]. However, side effects were noted from using the M6P-directed carriers, and in search for a better target, researchers have turned towards PDGFβR since it was also observed that it is specifically upregulated in liver fibrosis [191]. It is known that interferon γ (IFNγ) has exhibited side effects when administered systemically. The research group of Klaas Poelstra therefore created a fusion protein composed of IFNγ and PDGFβR bicyclic peptide. These fusion proteins were demonstrated to inhibit liver fibrogenesis in vivo, based on a significant increase in pSTAT1α activation, compared to the single unit of the fusion protein [192]. A drawback of these fusion proteins is their limited bioavailability, and therefore, a strategy to deposit them in the body is desirable. Van Dijk et al. thus developed biodegradable polymeric microspheres for the sustained release of such protein-based drugs [193].

**Figure 3:**
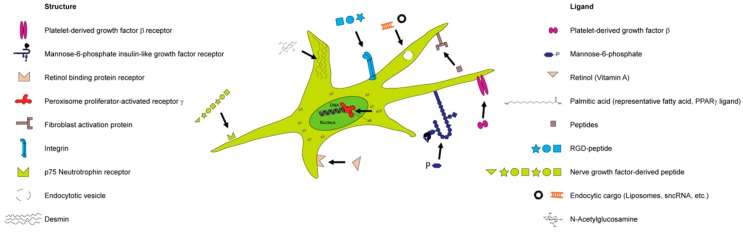
Ligand-based targeting of hepatic stellate cells. Hepatic stellate cells can be targeted based on their expression of receptors on their surface, in the cytoplasm, or inside the cell nucleus. The endocytic route allows the transport of HSC-directed sncRNA, small molecules, or liposomal carriers.

Integrins have also been explored as potential targeting moieties in this regard. Integrins fulfil important roles by connecting the intracellular cytoskeleton of cells to the ECM. Many integrins were reported to activate the transforming growth factor β (TGF-β), which is another critical factor in fibrogenesis [194]. However, direct inhibition of TGF-β is critical since it is involved in many anti-inflammatory processes. Thus, integrin targeting is in fact a type of indirect targeting of TGF-β. This was pioneered by Henderson et al. in 2013, showing that the pharmacological inhibition of αv integrins by a small molecule (CWHM-12) attenuated both liver and lung fibrosis [195]. Bansal and colleagues, in 2017, further explored the role of integrins in liver fibrosis. They observed that integrin α 11 (IαV) is critically involved in the regulation of the myofibroblast phenotype and that is colocalizes with α-smooth muscle actin-positive myofibroblasts. They further assumed that IαV apparently is involved in fibrogenic signaling and might act downstream of the hedgehog signaling pathway [196]. However, a drawback of integrin targeting is that integrins are important for many vital functions in the body. A scheme for several different ligands to target HSC receptors has been generated (Figure 3).

The hormone relaxin represents a potential anti-fibrotic ligand which binds to its receptor on HSC [197]. One of the important effects of relaxin is the widespread remodeling of the extracellular matrix, which includes altered secretion and degradation of its components [188]. The role of relaxin as an anti-fibrotic agent has been demonstrated in experiments on genetically engineered mice that are deficient in the relaxin gene. These mice spontaneously developed age-related fibrosis of the lungs, heart, skin, and kidneys [198]. It turned out that relaxin also affects the processes of fibrogenesis in the liver. Treatment with relaxin in rats caused acute changes in the microcirculation of the liver [198], and morphological changes were found in non-parenchymal sinusoidal cells [199]. The effects of relaxin were mediated by activation of the receptor for the peptide 1 family of relaxins (RXFP1), which is expressed predominantly in HSC in the liver [197]. Finally, using an in vivo model of experimental liver fibrosis, it was shown that relaxin prevented the content of liver collagen and was effective in treating established liver fibrosis [200]. Thus, relaxin has established itself as an active therapeutic agent in the treatment of liver fibrosis. However, free relaxin has a very short half-life due to its small size (6 kDa) and rapid renal clearance [201]. Frequent administration of relaxin in chronic fibrosis increases systemic vasodilation, which adversely affects the general condition of the body [199].

When studying the effects of relaxin on fibrosis, it was shown that relaxin conjugated nanoparticles significantly inhibited differentiation, TGF-β-induced migration, and contractile ability of human HSC in contrast to the control, where free relaxin was used; in addition, they drastically reduced collagen gel contraction with maximum inhibitory effects [176].

The evolving and expanding field of RNA technologies has a huge potential to bring up novel drugs. Recently, the first siRNA-based drug, Patisiran, was approved [202]. In the area of liver fibrosis research, Bangen and colleagues demonstrated that inhibiting the cell cycle protein cyclin E1 during liver fibrosis progression in CCl_4_-induced fibrosis significantly reduced disease severity [203]. RNA interference may also make use of nucleic acids to manipulate micro-RNA, which is known to also regulate the inflammatory processes in liver fibrosis [204]. Local release of therapeutic RNA might further improve efficiency of RNA interference technology.

In conclusion, the path towards an efficient treatment of fibrosis remains a rocky road equipped with many pitfalls. However, the high number of drugs evaluated for novel therapies is encouraging. Targeted drugs that might be further improved by magnetism may represent anti-fibrotic drugs of the upcoming generations.

## Figures and Tables

**Table 1:** Selected candidate drugs for treatment of hepatic injury and fibrosis.

Molecular Target	Compound	Effect
Apoptosis signal-regulating kinase 1 (ASK1)	Selonsertib (GS-4997)	oral bioavailable inhibitor of ASK1, thereby preventing the production of inflammatory and fibrotic acting cytokines
Hepatic metabolism	Obeticholic acid	synthetically modified bile acid and potent agonist of the farnesoid X nuclear receptor (FXR)
	Elafibranor	Orally administered drug acting on the 3 sub-types of PPAR (PPARα, PPARγ, PPARδ)
	Tropifexor	Investigational drug which acts as an agonist of the farnesoid X nuclear receptor (FXR)
	Cilofexor (GS-9674)	agonist of the farnesoid X nuclear receptor (FXR) which improves cholestasis and liver injury
	AKN-083	farnesoid X receptor (FXR) agonist
	INT-767	a dual agonist targeting the farnesoid X receptor (FXR) and the G protein-coupled bile acid receptor 1 (GPBAR1)
	Aramchol	An orally active fatty acid bile acid conjugate that inhibits stearoyl coenzyme A desaturase 1 (SCD1)
	Saroglitazar	Agonist of PPARα (and PPARγ)
	Lanifibranor	Orally administered drug acting on the 3 sub-types of PPAR (PPARα, PPARγ, PPARδ)
	Firsocostat (GS-0976)	Liver-targeted acetyl-CoA carboxylase (ACC) inhibitor
	PF-05221304	Liver-targeted acetyl-CoA carboxylase (ACC) inhibitor
Chemokine receptors	Cenicriviroc	blocks the chemokine receptors CC chemokine receptor 2 (CCR2) and CCR5
Caspases	Emricasan	Prevents cells death by inhibition of caspases
	VX-166	The drug has anti-apoptotic activity and prevents release of interleukins
	Nivocasan (GS-9450)	hepatoprotective activity preventing fibrosis and apoptosis
Fibroblast growth factor 21 (FGF21)	Pegbelfermin (BMS-986036)	PEGylated FGF21 analogue that improves metabolic parameters
Fibroblast growth factor 19 (FGF19)	Aldafermin (NGM282)	Synthetic FGF19 analogue preventing hepatic fat accumulation and liver damage
Glucagon-like peptide-1 receptor	Liraglutide	GLP-1 receptor agonist triggering insulin synthesis
	Semaglutide	GLP-1 receptor agonist triggering insulin synthesis

**Table 2:** Selected areas of applications for magnetic nanoparticles.

Role of MNP^1^	Area of Biomedical Application	Literature
Binding-mediated cell capturing	Cell isolation and separation	[141,143,144,145]
	Cell and tissue engineering	[146,147,148]
	Cell patterning and concentration	[149,150,151,152,153]
Mechanical cell control	Low-frequency magnetic field for cell destruction and induction of apoptosis	[78,155,156]
	Differentiation of stem cells, modulation of cell division and motility	[157,158,159]
	Fundamental study of macromolecules and cell‘s mechanical properties	[160,161]
Drug delivery	Magnetic fluid hyperthermia of cancer	[135,162,163]
	On-demand release of drugs via thermosensitive polymers or azo molecules from hybrid nanoplatforms	[133,164,165]
	Targeting or delivery of drug or genes immobilized on surfaces	[166,167,168,169]
Imaging applications	Reduction of T_1_ and T_2_ relaxation time of the water protons for the MRI-contrast	[66,124,170]
	Imaging and detection via a non-linear magnetic signal	[172,173]
	Improved detection of magnetic signals, imaging of liver fibrosis	[174,175]

^1^ MNP: Magnetic nanoparticle(s).
